# Low CD25 in ALK+ Anaplastic Large Cell Lymphoma Is Associated with Older Age, Thrombocytopenia, and Increased Expression of Surface CD3 and CD8

**DOI:** 10.3390/cancers17111767

**Published:** 2025-05-25

**Authors:** Shuyu E, L. Jeffrey Medeiros, Hong Fang, Shaoying Li, Guilin Tang, Sa A. Wang, Wei Wang, C. Cameron Yin, M. James You, Swaminathan P. Iyer, Luis Malpica, Lianqun Qiu, Zhenya Tang, Qing Wei, Pei Lin, Jie Xu

**Affiliations:** 1 Department of Hematopathology, The University of Texas MD Anderson Cancer Center, 1515 Holcombe Blvd Unit 72, Houston, TX 77030, USA; shuyu.e@bcm.edu (S.E.); ljmedeiros@mdanderson.org (L.J.M.); hfang@mdanderson.org (H.F.); sli6@mdanderson.org (S.L.); gtang@mdanderson.org (G.T.); swang5@mdanderson.org (S.A.W.); wwang13@mdanderson.org (W.W.); cyin@mdanderson.org (C.C.Y.); mjamesyou@mdanderson.org (M.J.Y.); lqiu@mdanderson.org (L.Q.); ztang@unmc.edu (Z.T.); qwei1@mdanderson.org (Q.W.); peilin@mdanderson.org (P.L.); 2 Department of Lymphoma, The University of Texas MD Anderson Cancer Center, Houston, TX 77030, USA; spiyer@mdanderson.org (S.P.I.); lemalpica@mdanderson.org (L.M.); 3 Department of Myeloma, The University of Texas MD Anderson Cancer Center, Houston, TX 77030, USA

**Keywords:** ALK+ anaplastic large cell lymphoma, CD25

## Abstract

Background/Objectives: Anaplastic lymphoma kinase (ALK)-positive (+) anaplastic large cell lymphoma (ALCL) is known to express CD25, but its significance has not been well studied. Methods: In the present study, we identified 54 ALK+ ALCL patients with CD25 results available and investigated the significance of CD25 expression levels. Results: Forty-two (78%) cases had high CD25 expressions, whereas low CD25 expressions were found in 12 (22%) cases. Compared with ALK+ ALCL patients with CD25-high neoplasms, patients with CD25-low neoplasms were older (median: 40 years vs. 29 years, *p* = 0.01) and more often had thrombocytopenia (40% vs. 0%, *p* = 0.02). Between CD25-low and CD25-high groups, other clinical features were similar to each other (all *p* > 0.05). CD25-low ALK+ ALCL cases showed higher frequency of surface CD3 (100% vs. 3%, *p* = 0.001) and CD8 (57% vs. 14%, *p* = 0.03). Fourteen of 47 (30%) ALK+ ALCL patients died (median follow-up time, 33.8 months; range, 0.3–382.8 months): 5 of 9 (56%) patients with CD25-low neoplasms and 9 of 38 (24%) patients with CD25-high neoplasms. Univariate analysis showed: (1) the OS of patients with CD25-low ALK+ ALCL was shorter than that of patients with CD25-high ALK+ ALCL (median: 72.2 months vs. undefined, *p* = 0.02); and (2) young (<30 years) patients with high CD25 expression had the best prognosis, with a long-term OS rate of 89%. However, in multivariate analysis, low CD25 expression did not significantly impact OS. Conclusions: most cases of ALK+ ALCL highly express CD25 which is a potential target for therapy. ALK+ ALCL with low CD25 expression is associated with older patient age and increased frequency of thrombocytopenia and surface CD3 and CD8 expression.

## Simple Summary

CD25 is a major lymphocyte growth factor and plays a critical role in the maturation, proliferation, and survival of T-cells. CD25 expression is often detected in ALK+ anaplastic large cell lymphoma (ALCL), but its significance is unclear. Here, we divided ALK+ ALCL cases into CD25-low and CD25-high groups based on their CD25 expression levels and compared the clinical and pathologic data between these two groups. We found that 78% of ALK+ ALCL cases showed high levels of CD25, suggesting that these patients may be treated with therapy regimens targeting at CD25. The ALK+ ALCL patients with low CD25 expressions were often older and more frequently had thrombocytopenia and surface CD3 and CD8 expression. Low CD25 expression had a significant, negative impact on overall survival in univariate analysis, but not in multivariate analysis.

## 1. Introduction

Anaplastic large cell lymphoma (ALCL) is a T-cell lymphoma which strongly and uniformly express CD30. ALCL is divided into ALK+ and ALK-negative subtypes. ALK+ ALCL is characterized by chromosomal translocations involving *ALK* at chromosome 2p23 and resultant ALK expression, representing approximately 10–15% of pediatric/adolescent and ~3% of adult non-Hodgkin lymphomas [1,2,3]. The 5-year survival rates of ALK+ ALCL patients range from 70% to 90%, which is much better than ALK-negative ALCL patients [4,5,6,7,8,9]. These neoplasms are morphologically heterogeneous and five morphologic patterns are recognized: common, small cell, lymphohistiocytic, Hodgkin-like, and composite patterns [10]. Some studies suggest an association between the small cell/lymphohistiocytic morphologic variants and a poorer clinical outcome [11].

CD25 is the α subunit of the IL-2 receptor (IL-2Rα). IL-2 is a major lymphocyte growth factor and plays a critical role in the maturation, proliferation, and survival of T-cells and T-cell-mediated immune response. Under normal physiologic conditions, CD25 is expressed at high levels by Treg (CD4+FoxP3+) cells [12]. CD25 also can be expressed by immature B cells, activated monocyte/macrophages, mast cells, and basophils [13,14,15,16]. It is well known that CD25 is expressed by the neoplastic cells of some hematopoietic/lymphoid neoplasms, such as ALCL, adult T-cell leukemia/lymphoma, hairy cell leukemia, B lymphoblastic leukemia/lymphoma (especially Ph+ or Ph-like type), acute myeloid leukemia, and systemic mastocytosis [17,18,19,20,21,22,23].

Most ALCL cases are positive for CD25, with the strongest expression in the ALK+ subset [24,25]. The levels of serum soluble IL-2R (CD25) levels are high in patients with ALK+ ALCL at diagnosis and these levels decrease in response to treatment [26]. Liang et al. examined CD25 expression in 88 pediatric ALK+ ALCL patients in a univariate analysis and found that patients with high CD25 ALK+ ALCL tended to have lower 5-year overall survival (OS) compared with those with low CD25 ALK+ ALCL; however, this difference did not reach statistical significance (*p* = 0.05) [25]. Therefore, the prognostic impact of CD25 expression in ALK+ ALCL patients is unsettled. In the present study, we examined 54 patients with ALK+ ALCL and compared the clinicopathologic features and outcome of patients with high versus low CD25 expression.

## 2. Materials and Methods

### 2.1. Case Selection

This study was performed under a protocol approved by the institutional review board. We reviewed the electronic medical records and collected the clinical data. We identified 54 ALK+ ALCL patients (2007–2018) who had CD25 expression results available. ALCL was diagnosed and subclassified according to the World Health Organization Classification (WHO; 5th edition) and the International Consensus Classification (ICC; 2022) [1,2].

### 2.2. Immunophenotypic Analysis

Immunohistochemical analysis was performed on formalin-fixed, paraffin-embedded (FFPE) tissue sections as described previously [27]. The antibodies used were specific for CD2 (Leica Biosystems Inc., Deer Park, IL, USA), CD3 (Dako, Carpinteria, CA, USA), CD4 (Cell Marque, Rocklin, CA, USA), CD5 (SP4; LabVision/NeoMarkers, Fremont, CA, USA), CD7 (Leica Biosystems Inc.), CD8 (Thermo Fisher, Waltham, MA, USA), CD20 (Dako, Carpinteria, CA, USA), CD25 (Leica Biosystems Inc.), CD43 (Dako, Carpinteria, CA, USA), CD45 (Dako, Carpinteria, CA, USA), ALK (Cell Signaling, Danvers, MA, USA), EMA (Leica Biosystems Inc.), granzyme B (Thermo Fisher, Waltham, MA, USA), and PAX5 (Transduction Laboratories, San Diego, CA, USA).

Flow cytometry immunophenotypic analysis was performed on cell suspensions of tissue specimens using either a FACScanto II or FACSCalibur cytometer (Becton Dickinson Biosciences, San Jose, CA, USA), as has been described previously [28]. The panel of monoclonal antibodies included reagents specific to CD2, CD3, CD4, CD5, CD7, CD8, CD10, CD25, CD30, CD45, CD56, T-cell receptor (TCR) alpha/beta, and TCR gamma/delta (Becton Dickinson Biosciences). ALCL cells were usually located in the monocyte gate, but in some cases were in the lymphocyte gate, CD45dim blast gate or para-granulocyte area, depending on their CD45 intensity and side scatter (SSC). Therefore, traditional lymphocyte gating strategies might miss the neoplastic cells. Back gating of CD30+ cells was used to help identify the neoplastic cells. For the patterns and intensity assessed by flow cytometry immunophenotyping, positivity for most markers was defined as expression in ≥20% of cells using a cut-off set by comparison to background fluorescence [22].

The results of CD25 expression by immunohistochemistry and/or flow cytometry were independently assessed by two pathologists (JX and SL). If ≥80% of lymphoma cells were positive for CD25 with a moderate to high expression strength by immunohistochemistry or flow cytometry, this case was determined as a CD25-high case. The cases that did not meet this criterion were determined as CD25-low cases.

### 2.3. Statistical Analysis

Statistical analyses were performed using the GraphPad Prism 10. Between the ALK+ ALCL patients with low vs. high CD25 expression, the clinical and pathologic data were compared using Fisher’s exact test. The date of initial diagnosis to the last follow-up or date of death was used to calculate the overall survival (OS). Kaplan–Meier method was used to analyze the OS, and the log-rank test was used to compare the OS in univariate analysis. We used Cox regression model and SPSS Statistics version 23 for statistical analysis in multivariate analysis. The result was considered statistically significant, if *p* is <0.05.

## 3. Results

### 3.1. Clinical Findings

This cohort of 54 ALK+ ALCL patients included 29 male and 25 female with a median age of 32 years (range, 5–74 years) at time of diagnosis. Forty-three of fifty-three (81%) patients had lymphadenopathy and twenty-nine of forty-six (63%) had extra-nodal involvement. Thirty-three of forty-four (75%) patients had stage III or IV disease and twenty-six of forty (65%) patients had B-type symptoms. Among these 54 ALK+ ALCL patients, 42 (78%) cases had high CD25 expressions and 12 (22%) had low CD25 expressions. The clinical features of these patients are summarized in Table 1.

The CD25-low group included four males and eight females with a median age of 40 years (range, 9–74 years) at time of diagnosis. In patients with CD25-low ALK+ ALCL, 7 of 11 (64%) had lymphadenopathy and 6 of 8 (75%) had extra-nodal involvement. Bone marrow was involved in three of seven (43%) patients and peripheral blood was involved in one of three (33%) patients. Seven of eight (88%) fully staged patients had stage III or IV disease. Six of seven (86%) patients had B-type symptoms. Four of five (80%) patients had leukocytosis (white blood cell count > 11.0 × 10^9^/L), and one of five (20%) patients had absolute lymphocytosis (lymphocyte count > 4.8 × 10^9^/L). Anemia was present in four of five (80%) patients and thrombocytopenia was identified in two of five (40%) patients. Three of five (60%) patients tested showed an elevated serum lactate dehydrogenase level. Only three patients had an International Prognostic Index (IPI) score available and no score was ≥three.

Compared to patients with CD25-high ALK+ ALCL, CD25-low patients were older (40 vs. 29 years, *p* = 0.01) and more frequently had thrombocytopenia (40% vs. 0%, *p* = 0.02). The clinical relevance of thrombocytopenia was uncertain due to the low number of patients with a platelet count available in CD25-low group (n = 5). There were no significant differences in other clinical features between patients with CD25-low versus CD25-high ALK+ ALCL (all *p* > 0.05; Table 1).

### 3.2. Pathologic Findings

**Figure 1:**
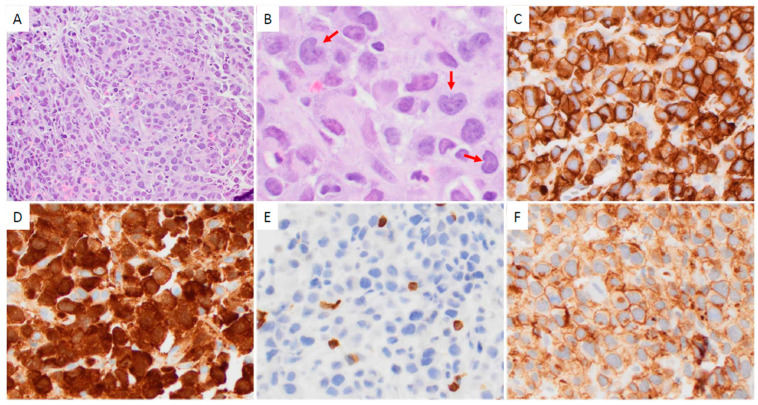
An ALK+ ALCL case with a common morphologic pattern and high CD25 expression involving lymph node. (**A**) At low magnification (×40), the nodal architecture is diffusely effaced by lymphoma cells. (**B**) At high magnification (×400), the lymphoma cells are large, with irregular nuclei and moderate amounts of cytoplasm. “Hallmark” cells with a kidney-shaped nuclei are present (red arrows). (**C**–**F**) The lymphoma cells are positive for CD30 ((**C**); ×200), ALK ((**D**), nuclear and cytoplasmic; ×200) and CD4 ((**F**); ×200), and are negative for CD3 ((**E**); ×200). (**A**,**B**) Hematoxylin-eosin stain. (**C**–**F**) Immunohistochemistry.

**Figure 2:**
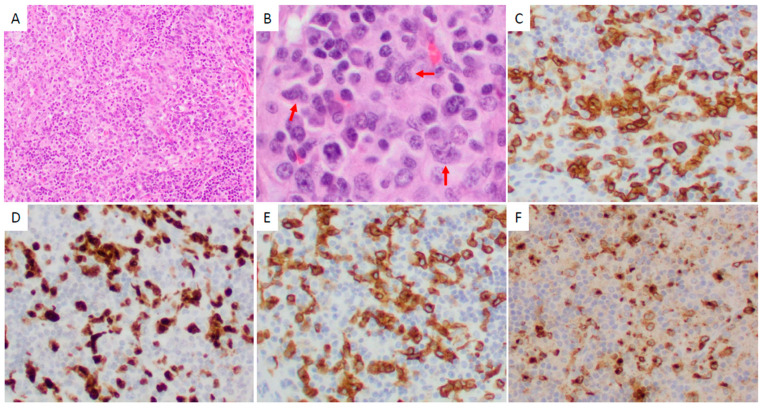
An ALK+ ALCL case with a small cell/lymphohistiocytic morphologic pattern and negative CD25 expression involving lymph node. (**A**) At low magnification (×100), a lymphohistiocytic infiltrate is identified. (**B**) At high magnification (×400), the lymphoma cells are intermediate in size, with irregular nuclei and a small to moderate amount of cytoplasm. “Hallmark” cells can be identified (red arrows). (**C**–**F**) The lymphoma cells are positive for CD30 ((**C**); ×200), ALK ((**D**), nuclear and cytoplasmic; ×200), CD3 ((**E**); ×200), and granzyme B ((**F**); ×200). (**A**,**B**) Hematoxylin-eosin stain. (**C**–**F**) Immunohistochemistry.

Thirty-four of forty-two (81%) CD25-high ALK+ ALCL cases showed a common morphologic pattern (Figure 1). Four of eight (50%) CD25-low cases showed small cell/lymphohistiocytic pattern (Figure 2). CD25-low cases tended to more frequently have a small cell/lymphohistiocytic pattern than CD25-high cases, but this difference was not significant (*p* = 0.08; Table 2).

**Figure 3:**
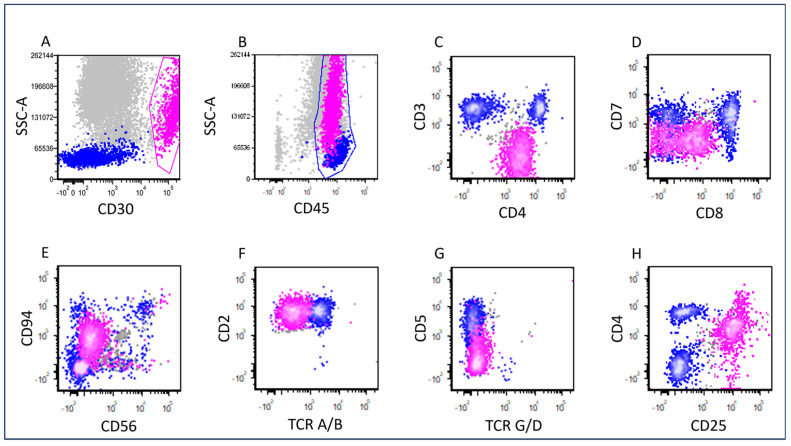
Flow cytometric immunophenotypic analysis of the case shown in Figure 1. The lymphoma cells (pink dots) are gated based on their strong CD30 expression (**A**). Back gating of the CD30+ cells on the SSC/CD45 plot shows that the lymphoma cells are positive for CD45 and are located at the para-granulocyte area (not the traditional lymphocyte gate) due to their high side scatter SSC (**B**). Therefore, the lymphocyte gate needs to be expanded to include all the lymphoma cells for accurate analysis. The lymphoma cells are positive for CD4 (decreased, (**C**)), CD8 (partial, (**D**)), CD94 (partial, (**E**)), CD2 (**F**), CD25 (**H**), and negative for surface CD3 (**C**), CD7 (**D**), CD56 (**E**), TCR alpha/beta (**F**), TCR gamma/delta (**G**), and CD5 (**G**). The blue dots represent background reactive T-cells.

**Figure 4:**
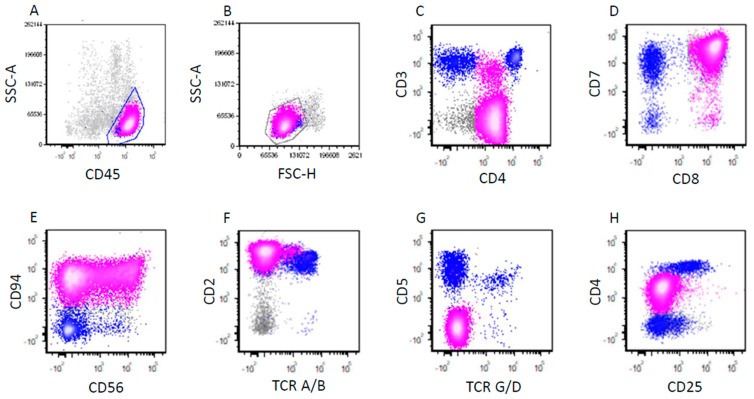
Flow cytometric immunophenotypic analysis of the case described in Figure 2. The lymphoma cells (pink dots) do not show significantly increased side scatter (SSC) (**A**) or forward side scatter (FSC) (**B**). The lymphoma cells are positive for CD45 (**A**), CD4 (decreased, (**C**)), CD7 (**D**), CD8 (**D**), CD94 (**E**), CD56 (partial, (**E**)), CD2 (**F**), and negative for surface CD3 (large subset), TCR alpha/beta (**F**), TCR gamma/delta (**G**), CD5 (**G**), and CD25 (**H**). A small subset of the lymphoma cells was dimly positive for surface CD3 (**C**). The blue dots represent background reactive T-cells.

**Figure 5:**
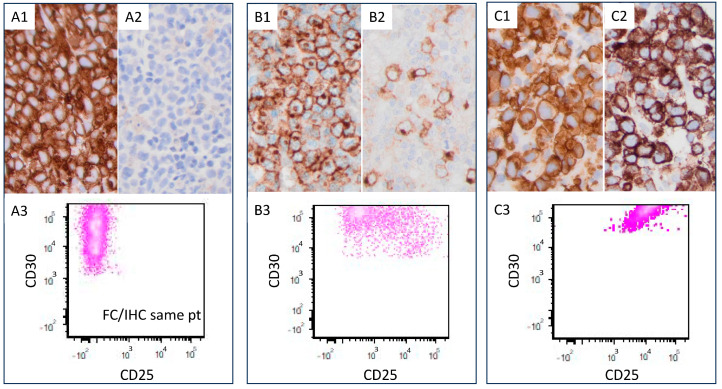
Three representative ALK+ ALCL cases with different CD25 expression levels. (**A**) In this case, the lymphoma cells are positive for CD30 (**A1**) and negative for CD25 (**A2**) by immunohistochemistry and flow cytometry (**A3**). (**B**) The lymphoma cells in this case are positive for CD30 (**B1**) and CD25 is <80% (**B2**) by immunohistochemistry and flow cytometry (**B3**). (**C**) In this case, the lymphoma cells are positive for CD30 (**C1**) and CD25 is >80% (**C2**) by immunohistochemistry and flow cytometry (**C3**).

The representative flow cytometric immunophenotypes of CD25-high and CD25-low ALK+ ALCL cases are shown in Figure 3 and Figure 4, respectively. CD25 expression determined by immunohistochemistry correlated with results determined by flow cytometry (Figure 5). The immunophenotypic features of these cases are summarized in Table 2. In the CD25-low group, all tested cases were positive for surface CD3 (n = six) and TIA1 (n = two). CD2 was positive in seven/eight (88%), CD45 in seven/eight (88%), EMA in seven/eight (88%), CD4 in six/eight (75%), granzyme B in three/four (75%), TCR alpha/beta in three/four (75%), CD5 in six/nine (67%), CD43 in four/six (67%), CD7 in four/seven (57%), CD8 in four/seven (57%), CD56 in one/three (33%), and BCL2 in one/four (25%). No tested cases were positive for CD15 (n = five) or TCR gamma/delta (n = four).

In the CD25-high group, all tested cases were positive for EMA (n = 16) and TIA1 (n = 7). Granzyme B was positive in 10/11 (91%), CD43 17/20 (85%), CD4 26/33 (79%), CD45 23/29 (79%), CD52 5/7 (71%), TCR alpha/beta 6/10 (60%), CD2 16/32 (50%), CD7 7/20 (35%), CD5 9/31 (29%), CD56 4/14 (29%), CD8 4/28 (14%), BCL2 1/9 (11%), CD15 1/18 (6%), and surface CD3 1/33 (3%). All nine cases tested were negative for TCR gamma/delta.

Compared with CD25-high ALK+ ALCL cases, CD25-low neoplasms were more often positive for surface CD3 (100% vs. 3%, *p* = 0.001) and CD8 (57% vs. 14%, *p* = 0.03). There were no significant differences in the expression of CD2, CD4, double CD4/CD8, CD5, CD7, CD15, CD43, CD45, CD56, BCL2, EMA, granzyme B, TIA1, TCR alpha/beta, TCR gamma/delta, and Ki67 between CD25-high versus CD25-low groups (all *p* > 0.05; Table 2).

### 3.3. Treatment and Response

Among patients with CD25-low ALK+ ALCL, six patients had chemotherapy and response information available (Table 1). All these six patients received cyclophosphamide, doxorubicin, vincristine, and prednisone (CHOP) or modified CHOP and four (67%) of them achieved complete response. Seven patients with CD25-low ALK+ ALCL had known status of stem cell transplantation (SCT) and three (43%) of them received SCT: one autologous followed by allogeneic, and two allogeneic.

Among patients with CD25-high ALK+ ALCL, 37 patients had chemotherapy information available (Table 1), and 26 (70%) patients received CHOP or modified CHOP. Thirty-six patients had treatment response information available, and thirty-one (86%) patients had complete response. Thirty-one patients with CD25-high ALK+ ALCL had known status of SCT, and thirteen (42%) patients received SCT: 11 autologous and 2 allogeneic. There were no differences in initial treatment or complete response rate between CD25-low versus CD25-high patients (all *p* > 0.05; Table 1).

### 3.4. Outcome

**Figure 6:**
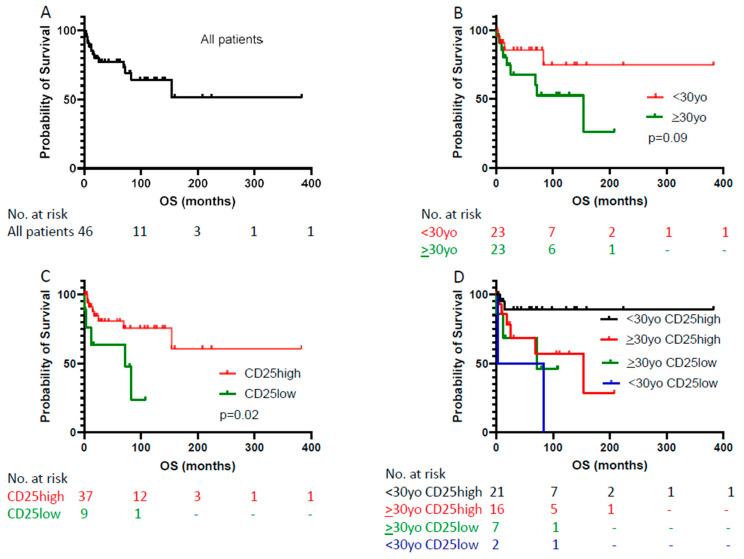
Assessment of prognostic significance of CD25 in patients with ALK+ ALCL. (**A**) The overall survival (OS) of all patients in this cohort. (**B**) Age (<30 vs. ≥30 years old) is not significantly associated with OS. (**C**) CD25 expression level is significantly associated with OS. (**D**) The prognostic significance of CD25 in younger (<30 years old) vs. older (≥30 years old) patients.

After a median follow-up of 33.8 months (range, 0–382.8 months), 14 of 47 (30%) patients died. The causes of death included lymphoma (n = eight), infection (n = three), lung cancer (n = one), graft versus host disease of lungs (n = one) and unknown (n = one). Five of nine (56%) patients with CD25-low ALK+ ALCL and nine of thirty-eight (24%) patients with CD25-high neoplasms died (Table 1; Figure 6A).

In univariate analysis, younger (<30 years old) patients tended to show longer OS than older (≥30 years old) patients, but the difference did not reach statistical significance (*p* = 0.09; Table 3; Figure 6B). Low CD25 expression was associated with shorter OS in ALK+ ALCL patients (*p* = 0.02; Table 2 and Table 3; Figure 6C). This association was still significant in younger (<30 years old) patients (*p* = 0.002; Figure 6D), but it was not seen in older (≥30 years old) patients (*p* = 0.61; Figure 6D). In the patient group with CD25-high ALK+ ALCL, patients, younger (<30 years old) patients showed longer OS than older (≥30 years old) patients (*p* = 0.04; Figure 6D). In the patient group with CD25-low ALK+ ALCL, there was no significant association between age (<30 vs. ≥30 years old) and OS (*p* = 0.56; Figure 6D). Among all ALK+ ALCL patients, those who were younger (<30 years) and had CD25-high neoplasm had the longest OS, with a long-term OS rate of 89% (Figure 6D). However, in multivariate analysis, CD25 expression level or age did not significantly affect OS (Table 3).

## 4. Discussion

ALCL is known to express CD25 in a subset of cases [29]. Juco et al. reported CD25 expression by flow cytometry in 14 of 16 (88%) ALCL cases (3 ALK+, 5 ALK-negative, 8 ALK status unknown) [24]. A recent study by Liang et al. confirmed that most ALCL cases are positive for CD25 by immunohistochemistry and that CD25 expression is expressed more strongly in ALK+ cases than in ALK-negative cases [25]. In the current study, 78% of ALK+ ALCL cases showed high CD25 expression whereas 22% of cases showed low CD25 expression, consistent with previous reports. Although ALK+ ALCL is genetically more homogenous than ALK-negative ALCL, with all cases carrying *ALK* rearrangement, their expression of antigens (such as CD25) still varies among cases. This may be related to different cell origins (CD4+ vs. CD8+ T-cells) and biological mechanisms (other non-*ALK* genetic/molecular alterations).

In addition to ALCL, CD25 is also expressed by the neoplastic cells of some other hematopoietic/lymphoid neoplasms such as acute myeloid leukemia and B lymphoblastic leukemia/lymphoma, especially the Ph+ or Ph-like type. In patients with acute myeloid leukemia and B lymphoblastic leukemia/lymphoma, high CD25 expression correlates with an adverse outcome [30,31,32,33,34,35]. To date, only one study in the literature investigated the possible prognostic impact of CD25 in ALCL. In univariate analysis of pediatric ALK+ ALCL patients, high CD25 expression was associated with poorer clinical outcome, but this association was of borderline significance (*p* = 0.05) [25]. Univariate analysis of our data showed overall that patients with CD25-low ALK+ ALCL had significantly shorter OS than patients with CD25-high ALK+ ALCL. Since CD25-low patients were older than CD25-high patients, we further investigated and excluded the possibility of age being a confounding prognostic factor. After stratifying the patients based on age, the association between CD25-low ALK+ ALCL and shorter OS was still significant in younger (<30 years) patients, but it was not seen in older (≥30 years) patients. ALK+ ALCL patients with young age (<30 years) and high CD25 expression had the best prognosis, with a long-term OS rate of 89%. However, in multivariate analysis, CD25 expression did not significantly impact OS, which may be partially due to the low number of CD25-low ALK+ ALCL patients in this cohort. In addition, the patients in this retrospective study received different treatment regimens and the cohort included both pediatric and adult patients, which may also limit the analysis of prognostic factors. Larger or prospective studies are needed to further investigate the prognostic significance of CD25 expression in ALK+ ALCL.

Although the CHOP/CHOP-like regimen has been the standard treatment for patients with ALK+ ALCL [36], recent years have witnessed the development of new therapeutic regimens. Brentuximab vedotin (BV), a chimeric monoclonal antibody-drug conjugate targeting CD30, has been integrated into front-line therapies for ALK+ ALCL. The BV-CHP regimen (CHOP with BV substituted for vincristine) was approved by the U.S. Food and Drug Administration (FDA) in November, 2018 for the treatment of adult patients with previously untreated systemic ALCL [37]. Another example is crizotinib, a first-generation ALK inhibitor, which was approved by the FDA in January, 2021 for treating pediatric and young adult patients with relapsed or refractory ALK+ ALCL [38].

The high expression level of CD25 in ALK+ ALCL makes it a potential therapeutic target. A CD25-targeting antibody-drug conjugate efficiently killed ALCL cells in vitro and in vivo [25]. IL-2, the ligand for CD25, accelerates ALCL cell growth and activates STAT1, STAT5, and ERK1/2 [25]. Ito et al. reported no IL-2 gene expression in cultured ALCL cells by RT-PCR, excluding the possibility of an IL-2 autocrine loop. Interestingly, immunostaining of ALCL tumor tissues showed IL-2 protein expression in background cells but not in lymphoma cells [39]. Similarly, Peuchmaur et al. found in patient specimens that reactive T-cells (not ALCL cells) were capable of synthesizing IL-2 mRNA [40]. These results suggest that a paracrine mechanism may be involved in the proliferation of these CD25+ ALCL cells and therapeutic approaches disrupting this loop may have potential in treating ALCL patients.

Anti-CD25 antibodies has been investigated as cancer therapy via at least two mechanisms: (1) directly targeting lymphoma cells expressing high level of CD25, such as ALCL [25,41]; (2) depleting Tregs to enhance the host anti-tumor immune response. Tregs express high levels of CD25 and play an important role in immune homeostasis, maintaining immune tolerance to self- and non-self-antigens. Given their role in tumor immune escape, Tregs are potential targets for anti-tumor immunotherapy. In earlier years, anti-CD25 treatment of patients with ALCL only produced a transient response, lasting from one to more than eight months after anti-CD25 therapy [42,43]. The treatment responses of more recent CD25-targeting therapy in T-cell lymphoma/leukemia patients are more promising. In patients with early- and late-stage CD25+ CTCL, a recombinant fusion protein targeting IL-2R had a significant and durable effect on overall response rate and progression-free survival [44]. In patients with refractory ALK+ and ALK-negative ALCL, treatment with daclizumab (anti-CD25) has been successful, leading to clinical remission for at least 12 months in ALK+ and 45 months in ALK-negative ALCL, respectively [45,46].

ALCL, particularly ALK+ type, is often negative for surface CD3 expression [24]. Shen et al. reported that 39% of ALK+ and 50% of ALK-negative ALCL cases were positive for surface CD3 by flow cytometry [28]. In the present study, 18% (7/39) of ALK+ ALCL cases were positive for surface CD3, in keeping with the literature. Interestingly, among the seven cases with positive surface CD3, six (86%) cases were from the CD25-low group. Therefore, the rate of surface CD3 expression was significantly higher in the CD25-low than the CD25-high groups (100% vs. 3%), suggesting an inverse relationship between CD25 and surface CD3 expression.

Most ALCL cases express CD4, but a small subset of ALCL cases express CD8. The reported rates of CD8 expression in ALCL vary in the literature up to 24% [11,28,47,48]. CD8 was assessed by Shen et al. in 158 patients with systemic ALCL: CD8 was positive in 19% of ALK+ and 14% of ALK-negative neoplasms [28]. In the present study, 23% (eight/thirty-five) of ALK+ ALCL showed CD8 expression, consistent with previous reports. The CD25 expression rate and level are usually higher in CD4+ versus CD8+ T-cells. Vargas et al. reported CD25 expression in 54.8% of CD4+FoxP3+ Treg cells, 7.5% of CD4+FoxP3− cells, and 1.9% of CD8+ cells [12]. The authors also showed that the level of CD25 expression, as assessed by mean fluorescence intensity (MFI), was significantly higher on CD4+FoxP3+ Treg cells relative to CD4+FoxP3− and CD8+ T-cells (mean MFI: Treg cells = 190.0, CD4+FoxP3− cells = 34.5, and CD8+ cells = 17.9). We found that CD25-low ALK+ ALCL cases had a higher CD8 expression rate than CD25-high cases. About 30% of the CD25-low cases were CD4+CD8+, suggesting that most CD25-low cases may be derived from CD4−CD8+ T-cells whereas a small subset may be derived from CD4+CD8+ T-cells.

## 5. Conclusions

Most cases of ALK+ ALCL highly express CD25 indicating that CD25 is a potential therapeutic target in ALCL patients. Low CD25 expression identified non-typical ALK+ ALCL cases characterized by older age, thrombocytopenia, and increased surface CD3 and CD8 expression. Low CD25 expression had a significant impact on OS in univariate analysis, but not in multivariate analysis. Our data suggest that assessing CD25 in ALK+ ALCL cases may be potentially useful in guiding targeted therapy, but its prognostic significance needs to be further investigated in larger studies.

## Figures and Tables

**Table 1:** Clinical features of patients with CD25-high vs. CD25-low ALK+ ALCL.

	CD25-High (n = 42)			CD25-Low (n = 12)			*p* Value
	Positive	Total Tested	%	Positive	Total Tested	%	
Clinical features (at diagnosis)							
Male/female	25/17 (1.5:1)			4/8 (0.5:1)			0.19
Age (median, range)	29 (5–58)			40 (9–74)			0.01
Nodal presentation	36	42	86	7	11	64	0.19
Extra-nodal involvement	23	38	61	6	8	75	0.69
BM+	8	36	22	3	7	43	0.35
PB+	1	8	13	1	3	33	0.49
Stage III–IV	26	36	72	7	8	88	0.66
B symptoms	20	33	61	6	7	86	0.39
Laboratory at diagnosis							
Elevated WBC (>11.0 × 10^9^/L)	9	26	35	4	5	80	0.13
Absolute lymphocytosis (>4.8 × 10^9^/L)	2	24	8	1	5	20	0.45
Absolute lymphopenia (<1.0 × 10^9^/L)	4	24	17	1	5	20	1
Anemia (M < 14; F < 12 g/dL)	18	26	69	4	5	80	1
Thrombocytopenia (<140 × 10^9^/L)	0	26	0	2	5	40	0.02
Elevated LDH *	11	23	48	3	5	60	1
IPI ≥ 3	6	28	21	0	3	0	1
Initial treatment							
CHOP or modified CHOP	26	37	70	6	6	100	0.31
Other treatment (other chemo, or RT)	11	37	30	0	6	0	
Initial CR	31	36	86	4	6	67	0.26
SCT performed	13	31	42	3	7	43	1
Outcome							
Alive	29	38	76	4	9	44	0.1
Dead	9	38	24	5	9	56	
OS (months) (median, range)	40.0 (0–382.8)			14.1 (0.3–108.1)			0.02

Abbreviations: BM, bone marrow; PB, peripheral blood; WBC, white blood cell; LDH, lactate dehydrogenase; IPI, International Prognostic Index; CHOP, cyclophosphamide, adriamycin, vincristine, prednisone; RT, radiation therapy; CR, complete response; SCT, stem cell transplant; OS, overall survival. *, >618 U/L before 26 April 2018; >214 U/L on or after 26 April 2018.

**Table 2:** Pathologic features of CD25-high vs. CD25-low ALK+ ALCL Cases.

	CD25-High (n = 42)			CD25-Low (n = 12)			*p* Value
	Positive	Total Tested	%	Positive	Total Tested	%	
Morphology							
Common pattern	34	42	81	4	8	50	0.08
SC/LH pattern	8	42	19	4	8	50	
Immunophenotype							
CD2+	16	32	50	7	8	88	0.11
Surface CD3+	1	33	3	6	6	100	0.001
CD4+	26	33	79	6	8	75	1
CD8+	4	28	14	4	7	57	0.03
CD4+CD8+	2	27	7	2	7	29	0.18
CD5+	9	31	29	6	9	67	0.053
CD7+	7	20	35	4	7	57	0.39
CD15+	1	18	6	0	5	0	1
CD43+	17	20	85	4	6	67	0.56
CD45+	23	29	79	7	8	88	1
CD52+	5	7	71	1	1	100	1
CD56+	4	14	29	1	3	33	1
BCL2+	1	9	11	1	4	25	1
EMA+	16	16	100	7	8	88	0.33
Granzyme B+	10	11	91	3	4	75	0.48
TIA1+	7	7	100	2	2	100	1
TCR A/B+	6	10	60	3	4	75	1
TCR G/D+	0	9	0	0	4	0	1
Mean Ki67 (%)	85			81			0.55

Abbreviations: SC/LH, small cell/lymphohistiocytic; TCR A/B, T cell receptor alpha/beta; TCR G/D, T cell receptor gamma/delta.

**Table 3:** Univariate and multivariate analysis of age and CD25 expression level on overall survival.

	Univariate Analysis			Multivariate Analysis		
	*p* Value	HR	95% CI	*p* Value	HR	95% CI
Age ≥ 30 years old	0.09	2.67	0.90–7.94	0.16	2.40	0.72–8.10
Low CD25 expression	0.02	3.41	0.76–15.33	0.15	2.34	0.73–7.51

Abbreviations: HR, hazard ratio; CI, confidence interval.

## Data Availability

The datasets used and/or analyzed during the current study are available from the corresponding author on reasonable request.
